# Urinary Incontinence in Parous Women Practicing Non-Extreme Competitive Sports Compared to the General Population

**DOI:** 10.3390/jcm12082803

**Published:** 2023-04-10

**Authors:** Masha Ben Zvi, Maya Arad Cohen, Matan Friedman, Hadas Ganer Herman, Eran Weiner, Shimon Ginath

**Affiliations:** Urogynecology and Pelvic Floor Unit, Department of Obstetrics and Gynecology, Edith Wolfson Medical Center, Affiliated to the Sackler Faculty of Medicine, Tel Aviv University, Holon 5822012, Israel

**Keywords:** urinary incontinence, stress urinary incontinence, physical activity

## Abstract

Introduction and objective: This cross-sectional study aimed to compare the prevalence of urinary symptoms in physically active females to the general population represented by medical staff. Materials and methods: We conducted a survey utilizing the UDI-6 questionnaire on women playing catchball for at least one year and training twice a week or more in an official Israeli competitive league. The control group consisted of women practicing medicine (physicians and nurses). Results: The study group consisted of 317 catchball players and the control group consisted of 105 medical staff practitioners. Both groups were similar in most of the demographic characteristics. Urinary symptoms represented by UDI-6 scores were higher in women in the catchball group. Frequency and urgency symptoms were common in women playing catchball. Stress urinary incontinence (SUI) was insignificant between the groups (43.8% in the catchball group and 35.2% in the medical staff group, *p* = 0.114). However, severe symptoms of SUI were more common in catchball players. Conclusions: The rates of all urinary symptoms were higher in in catchball players. SUI symptoms were common in both groups. However, severe symptoms of SUI were more common in catchball players.

## 1. Introduction

Urinary incontinence (UI) is an involuntary loss of urine. According to International Urogynecological Association (IUGA) and the International Continence Society (ICS), UI can be divided into three subtypes: stress urinary incontinence (SUI), urgency urinary incontinence (UUI), and mixed urinary incontinence (MUI) [1]. Stress urinary incontinence is defined as the involuntary leakage of urine with physical exertion, coughing, laughing, or sneezing. Urine leakage occurs due to increased intraabdominal pressure and an inability to maintain the normal pressure gradient between the bladder and urethra. SUI is more common among women and is often associated with a history of pelvic floor weakness secondary to vaginal delivery, trauma, or surgery. Urgency urinary incontinence (UUI) is the complaint of involuntary leakage of urine accompanied by or immediately preceded by urgency. The underlying mechanism associated with UUI is detrusor overactivity and most commonly does not have an identifiable cause but can result from neurogenic or myopathic bladder dysfunction. Overactive bladder (OAB) is a term commonly used to describe the symptoms associated with urinary urgency incontinence and has recently become the preferred term to describe the spectrum of urgency urinary disorders. A diagnosis of OAB does not require the presence of UI and must be made in the absence of a urinary tract infection (UTI). Patients who report voiding greater than eight times in 24 h meet the accepted frequency threshold for OAB. Their symptoms are often accompanied by nocturia, which occurs in the absence of a UTI or other urinary pathology. OAB can be further subdivided into “wet” and “dry” to describe those with or without associated UI [2]. Overflow urinary incontinence is the involuntary loss of urine associated with bladder overdistention. The underlying cause of overflow incontinence is usually related to either inadequate detrusor contraction or bladder outlet obstruction, secondary to severe pelvic organ prolapse (POP), excessive tension after a UI surgical procedure, or detrusor underactivity of unknown etiology. Patients can also present with combined symptoms of both stress and urgency UI, defined as mixed urinary incontinence.

Urinary incontinence is more common in women than in men and may affect women of all ages. Prevalence rates in women between 15 and 64 years vary between 10% and 55%. Only one-quarter of these women seek help for the problem, but still the approximate annual cost of the condition in the US has been estimated at $US11.2 billion in the community and $US5.2 billion in nursing homes (1996 values) [3].

Stress urinary incontinence (SUI) is the most common subtype of UI and is defined as the involuntary loss of urine during physical exertion, increasing abdominal pressure [4]. It may occur during physical activity and everyday events such as coughing, sneezing, or laughing [5]. The recently reported SUI prevalence in the USA was 46% [6]. The main risk factors for SUI are pregnancy, vaginal delivery, and chronic medical conditions that cause an increase in intra-abdominal pressure, such as constipation and cough.

High-impact activity has been associated with higher rates of urinary incontinence [7,8]. UI occurs in about 20–50% of female athletes of all ages and parity status. It also occurs in about 40% of younger nulliparous athletes and is more prevalent in high-impact sports, where both feet are off the ground simultaneously, as it involves an abrupt repeated increase in intra-abdominal pressure [7,9]. UI is associated with medical and psychological morbidity that negatively influences the quality of life (QoL), which can influence athletic performance [10].

Two opposing hypotheses have been suggested for modifications of the pelvic floor in elite athletes: (i) female athletes have strong pelvic floor muscles (PFM); and (ii) female athletes may overload, stretch, and weaken the pelvic floor [3]. The rationale for hypothesis one was that any physical activity that increases abdominal pressure would lead to a simultaneous or pre-contraction of the PFM, and the muscles would be trained. Based on this assumption, general physical activity would prevent and treat SUI. However, women leak during physical activity and report worse leakage during high-impact activities. No sports involve a voluntary contraction of the PFM. Many women do not demonstrate an effective simultaneous or pre-contraction of the PFM during increased abdominal pressure.

Regarding hypothesis two, female athletes may overload, stretch, and weaken the pelvic floor. Heavy lifting and strenuous work have been listed as risk factors for developing pelvic organ prolapse and SUI. Repeated increases in abdominal pressure due to hard manual work and chronic cough might damage the cardinal and uterosacral ligaments, PFM, and the connective tissue of the perineum.

Regular physical activity is associated with lower mortality and decreased risk of developing chronic diseases such as diabetes, hypertension, and colon cancer than inactivity [11]. It positively impacts muscle and bone strength, improves psychological well-being, and decreases body fat [11]. Urinary incontinence is perceived as a barrier to exercise, particularly by women with more severe leakage. Given the many benefits of exercise, it is prudent to know the extent of the problem to remove possible obstacles to exercise, such as SUI. SUI implies that urine loss occurs during increases in abdominal pressure, especially during physical activity. Thus, sedentary women less exposed to physical exertion may not manifest SUI, although the underlying condition might be present. In an Australian study, which included 4556 women, 46% stopped exercise they had previously participated in due to their pelvic floor symptoms. Urinary incontinence had the largest impact; 41% with UI, followed by 37% with pelvic organ prolapse and 26% with anal incontinence stopped at least one form of exercise. Forty-two percent of women who experienced symptoms in high-impact sports stopped participation (versus low-impact: 21%). Sports commonly ceased include volleyball (63%), racquet sports (57%), and basketball (54%). Exercise cessation was reported amongst younger (18–25 years: 35%) and nulliparous women (31%). Common exercise modifications included lowering the intensity (58%) or frequency (34%) of participation or changing to a low-impact form of sport/exercise (45%) [12].

In the last two decades, a newly developing field of competitive sport for women only has evolved in Israel—catchball. Catchball is a team sport derived from volleyball in which the ball is caught and thrown rather than hit. The sport was started in Israel, and was designed to be a simple version of volleyball for women to play. It is now one of the most popular sports in Israel played by female adults. It is the fast-growing sport in Israel, third in the number of registered players after soccer and basketball, with its association and various competitive leagues. The rules of catchball are very similar to volleyball though, by allowing the players to catch the ball before throwing it to another player or over the net, it is easier to play and learn. Like volleyball, it is a high-impact physical activity where both feet are off the ground simultaneously, which involves an abrupt, repeated increase in abdominal pressure, except players can catch and hold the ball for less than one second. Additionally, most players are in their forties and parous women. Although it was reported that professional players in volleyball had a higher rate of urine loss [13], there is no information regarding the catchball players.

The population of parous women practicing competitive sports has never been studied concerning urinary symptoms. We conducted this cross-sectional study to improve our knowledge and understanding of stress incontinence in this particular cohort and better understand urinary incontinence and exercise in a unique population of parous women.

Our aims were to describe the prevalence of urinary incontinence in physically active parous women practicing competitive sports compared to the general population represented by medical staff, physicians and nurses, and to estimate whether an association exists between exercise and leakage severity. The primary outcome was to evaluate the UDI-6 between the groups.

## 2. Materials and Methods

Women over 25 years who played catchball in a competitive league for at least a year, practiced at least twice a week, and were willing to participate were included. In the control group, the inclusion criteria were age over 25 years. Pregnant women and those with previous SUI treatment were excluded from both groups.

After obtaining an institutional ethics committee approval, a self-administered questionnaire was developed to assess the prevalence of incontinence in catchball players playing in competitive leagues practicing at least two times a week or more. The questionnaire contained three sections (first: demographics, gynecologic, and obstetric history; second: sports-related data; and third: UDI-6 questionnaire). The questions involved information about demographic data, body mass index (BMI), parity, maximal birth weight, route of delivery, hormone use, other medications, and surgery involving the bladder, rectum, or uterus. Sports-related data included duration and amount of time spent in catchball practices and games per week, the years they have practiced this sport, and practicing in other types of exercises. Symptoms of urinary incontinence and were evaluated using questions 15–20 on the Pelvic Floor Distress Inventory-Short Form (PFDI-20) questionnaire (UDI-6 = Urinary Distress Inventory) [14]. A positive answer to questions 15–20 on the PFDI-20 questionnaire (UDI-6 = Urinary Distress Inventory) of “moderately” (3) or “quite a bit” (4) was used to define clinically significant lower urinary tract symptoms.

With the cooperation of the catchball association, the questionnaires were distributed to catchball teams during the competitions or via the internet. A similar questionnaire was used in the control group, which included female physicians, midwifes, and nurses in our hospital, leaving out the questions regarding catchball.

The primary study outcome was the difference in UDI-6 scoring between the groups and the secondary outcomes were the patient-reported symptoms according to each question separately.

Statistical analysis: Continuous variables were calculated as mean ± SD or median and range, as appropriate. Categorical variables were calculated as rate (%). *t*-test was used to compare continuous parameters, and chi-square or Fisher’s test was used to analyze categorical variables. Multiple linear regression analysis was performed to control confounders that can influence the groups’ results regarding urinary symptoms. Statistical significance was considered at *p* < 0.05. Sample size calculation was based on the primary outcome, the difference in UDI-6 scoring between the groups. Since no previous study used such scoring to compare groups of athletes and controls, we used convenience sampling on the current clinical outcome. After we analyzed the data, we validated our results with a power calculation as follows. For the primary outcome (UDI-6), we evaluated 317 study subjects and 105 control subjects. The response within each subject group was normally distributed with a standard deviation of 6. Since the actual difference in the study and control means is 6, we can reject the null hypothesis that the population means of the experimental and control groups are equal with a probability (power) of 1.000, and the Type I error probability associated with this test of this null hypothesis is 0.05. Data were analyzed using SPSS statistical software version 23 (SPSS, Inc., Chicago, IL, USA).

## 3. Results

During the study period, 321 catchball players and 107 controls completed the questionnaire. Four women were excluded from the study group (two played less than a year, two had previous trans obturator tape (TOT) insertion), two women in the control group were excluded (previous TOT insertion). The control group comprised 83 (79%) nurses and midwives, and 22 (21%) physicians. The average age in the study and the control group was 42.6 (range 27–60) and 41.0 (range 25–59), respectively. The demographic characteristics of both groups are shown in Table 1. Except parity there was no difference between the groups regarding age, BMI, maximal birth weight, type of delivery, additional training, menopause, and oral contraceptive use.

Catchball players’ and medical staff’s answers to UDI-6 questions are presented in Table 2. Urinary symptoms represented by UDI-6 scores were higher in women in the catchball group. Frequency and urgency symptoms were also common in women playing catchball. Stress urinary incontinence (SUI) was insignificant between the groups (43.8% in the catchball group and 35.2% in the medical staff group, *p* = 0.114). However, severe SUI was more common in catchball players (Table 2). The results between the groups were the same even following controlling the following parameters: age, parity, BMI, maximal birth weight, cesarean deliveries, and additional training by multiple linear regression analysis.

## 4. Discussion

The results of the present study demonstrated a high prevalence of urinary tract symptoms in the catchball group of patients. The difference between the groups was significant in symptoms of urgency and frequent urination. Regarding SUI symptoms, there was no difference between the groups (44% and 35%, respectively). However, women in the catchball group had a higher rate of significant symptoms of SUI (27.1% and 17.1%, respectively).

UI has been defined as any involuntary leakage of urine [1]. However, some authors have chosen to restrict prevalence figures according to the frequency of involuntary urinary leakage—for example, based only on daily, weekly, monthly, or annual urinary leakage. The prevalence of stress incontinence in the general population reported in various studies has a wide range, depending on age and definition of incontinence. Thus, for the reasons given earlier, it is difficult to compare the results of different population studies.

In a review of population studies from numerous European countries [15], the prevalence of any UI is in the range of 25–45%. In an extensive cross-sectional web-based survey of over 15,000 women, the prevalence of stress urinary incontinence at least “sometimes” was as high as 31.8% [16]. Current data describe a wide variation in prevalence rates of incontinence in women. However, International Consultation on Incontinence reports that 10% of all adult women report leakage at least weekly, and 25–45% experience occasional leakage [17].

The age of onset may be an essential factor in the type of UI experience. Fultz et al. found that the median age of American women reporting stress incontinence was 48 years [18]. In our study, the mean age for both groups was forty-two.

SUI in athletes is related to how frequently athletes are subjected to increased intrabdominal pressure, which is caused by a contraction of the abdominal muscles in high impact activities. Strenuous activity that involves intra-abdominal pressure can overload and chronically damage the perineum, including the periurethral striated muscle, thus decreasing the contraction strength of the pelvic floor muscles and increasing the risk of SUI. Another factor is muscle fatigue, which occurs during physical activity when type 2 fibers are recruited. In parous women there is anatomic damage to the fascia supporting the urethra that adds to the pathophysiology of stress urinary incontinence [19].

Many studies report the prevalence of occupation-related incontinence in elite athletes. In a review of 22 studies, which included 7507 women aged 12 to 69 years who practiced high- or moderate-impact activities involving jumping, fast running, and rotational movements, the prevalence of UI varied from 5.6% in low-impact activity to 80% in trampolining. High-impact activities showed a 1.9-fold prevalence over medium-impact activities [20].

In another review of nine studies that included reports of UI in female athletes who practiced in different sports, the prevalence of UI was 25.9% and of SUI was 20.7%. The most prevalent high-impact sport was volleyball, with a value of 75.6% [21].

One of the main findings in the current study is the high rate of frequency and urgency symptoms of the catchball players. It was speculated that high-impact sports were more frequently associated with incontinence, while low-impact sports were more frequently associated with lower urinary tract symptoms, such as urgency and frequency. Simeone et al. assessed the prevalence of LUTS and incontinence among 623 Italian female athletes practicing different sports with a mean age of 26. Amateur athletes were more affected by urinary frequency than competitive athletes. However, the prevalence of stress incontinence was 9.1% and half of them suffered from incontinence only during sports activity, mainly high-impact sports such as volleyball or trampoline [22].

A few studies compared the prevalence of SUI in female athletes to the general population. Caylet et al. assessed 157 athletes and 426 controls. Urinary incontinence was reported in 28% of the athletes’ group and 9.8% of the non-athletes. The non-athletes group was divided into physically active and non-physically active, and the numbers of SUI in both groups were similar [7]. KARI BØ performed a two-stage study to assess the prevalence among athletes and controls. No significant difference in the prevalence of stress urinary incontinence (SUI) in the athletes and controls was observed, 41% and 39%, respectively. However, we should consider the parity of the groups; 4% of the athletes and 33% of the controls had delivered, which may mask the analysis of the results [23].

Catchball is very similar to volleyball in many aspects. Da Silva Pereira et al. [13] compared 45 professionals to 30 amateur volleyball players with regard to stress urinary incontinence during competition. They evaluated urine loss during competition by self-report questionnaire and by a pad test. Day to day incontinence was evaluated by UDI-6. Their results showed approximately half of both the professional and the amateur players had symptoms of urine loss during competitions. They observed that self-report of UI during competition was common among both groups; however, objective urine loss represented by pads was significantly higher among professional athletes.

The control group of our study included medical staff, mainly nurses and midwives. Similar findings of urinary tract symptoms were also found in previous studies involving medical staff. The prevalence of UI in a USA nurses’ health study was 38% [24] and 56% of women reported SUI symptoms [25]. In the Japan Nurses’ Health Study, in women aged 27–82 years, the prevalence of SUI was 13.9%, OAB was 9.5%, and MUI was 2.1% [26].

There were several weaknesses in our study. The results are based on a validated questionnaire and do not include a physical examination or the quantification of urine loss, which may have shown slightly different objective results. However, it is not relevant for the treatment since we need to treat objective pathologies that are not bothersome for the patient. Also, evaluation of the groups in a larger sample size would be desirable, especially for regression analysis.

The strength of our study is the unique population we studied. Most were parous women, and the average age was forty-two. Age and parity are the main factors in the predisposition to UI. Hence, studying this population is indispensable to accurately estimating such an entity’s prevalence. Another factor is the rate of disclosure. The survey we conducted was anonymous. Herein the results were more accurate.

Regarding the association between competitive physical activity and stress incontinence, most studies were conducted on elite athletes who do not represent most physically active women. To the best of our knowledge, the data we presented describe all aspects of urinary tract symptoms in middle-aged women engaged in non-extreme competitive sports for the first time.

Currently, exercise is the normality rather than the exception for many women. However, sometimes UI may be perceived as caused by exercise and a barrier to engaging in physical activity. Our study suggests that the problem is broad within the female parous population, whether they practice sports or not. Many women perceive incontinence as an inevitable part of aging or childbirth; as such, they feel it is something to be tolerated, and many are often embarrassed or ashamed of this problem. Many are unaware that SUI can be treated with pelvic floor muscle exercise or surgery. This study should demonstrate how common this problem is and, instead, should push towards an awareness campaign so more women will seek help and not give up physical activity, so beneficial for physical and mental health.

## 5. Conclusions

The rates of urinary symptoms were higher in catchball players, mainly symptoms of urgency and frequent urination. SUI was common in both groups. However, severe symptoms of SUI were more common in catchball players. The fact that SUI is common in both groups implies it should not be a barrier for women to avoid physical activity. Instead, it should trigger an awareness campaign to help improve women’s quality of life.

## Figures and Tables

**Table 1:** Demographic characteristics.

	Catchball Players (*n* = 317)	Controls (*n* = 105)	*p*
Age (years)	42.6 ± 5.9	41.0 ± 9.0	*0.026*
Parous	303 (95.6%)	88 (83.8%)	*<0.001*
Parity	2.6 ± 1.0	2.0 ± 1.3	*<0.001*
BMI	23.9 ± 4.6	24.7 ± 4.7	*0.351*
Maximal birth weight (g)	3509.0 ± 538.4	3432.6 ± 421.3	*0.069*
Cesarean deliveries	76 (24.0%)	19 (18.0%)	*0.211*
Additional training	117 (36.9%)	47 (44.8%)	*0.127*
Menopause	43 (13.6%)	19 (18.1%)	*0.256*
Oral contraceptive use	29/274 (10.6%)	11/86 (12.7%)	*0.570*

Mean ± SD; BMI = Body mass index.

**Table 2:** UDI 6 outcome.

	Catchball Players (*n* = 317)	Controls (*n* = 105)	*p*	*p^#^*
Q1. Do you usually experience frequent urination?	89 (28.1%)	18 (17.1%)	*0.026*	*0.017*
Q2. Do you usually experience incontinence related to a sense of urge?	75 (23.7%)	13 (12.4%)	*0.014*	*0.011*
Q3. Do you usually experience incontinence related to sneezing, coughing, or laughing?	139 (43.8%)	37 (35.2%)	*0.121*	*0.110*
Q3. Do you usually experience incontinence related to sneezing, coughing, or laughing? (moderately and or quite a bit)	86 (27.1%)	18 (17.1%)	*0.04*	*0.069*
Q4. Do you usually experience leakage of small amounts of urine?	98 (30.9%)	22 (21.0%)	*0.050*	*0.008*
Q5. Do you usually experience trouble emptying your bladder?	21 (6.6%)	6 (5.7%)	*0.741*	*0.399*
Q6. Do you usually experience discomfort or pain in your lower abdomen or genital area?	38 (12.0%)	15 (14.3%)	*0.538*	*0.668*
UDI-6 *	17.8 ± 21.2	11.6 ± 15.3	*0.011*	*0.003*

UDI-6 = Urinary Distress Inventory; * mean ± SD; *p*^#^ = Multiple linear regression analysis controlling age, parity, BMI, maximal birth weight, cesarean deliveries, and additional training.

## Data Availability

The data supporting reported results are available upon request.
